# The effect of blood pressure variability on the prognosis of patients with acute cerebral hemorrhage: Possible mechanism

**DOI:** 10.3389/fnins.2022.1035061

**Published:** 2022-12-05

**Authors:** Xiangrong Sun, Xinyue Jv, Qi Mi, Qian Yang, Tao Chen, Guohui Jiang

**Affiliations:** ^1^Department of Neurology, Affiliated Hospital of North Sichuan Medical College, Nanchong, Sichuan, China; ^2^Department of Neurology, The Third Affiliated Hospital of Chongqing Medical University, Chongqing, China; ^3^Wusheng County People’s Hospital, Wusheng, Sichuan, China; ^4^Department of Neurosurgery, Guangyuan Central Hospital, Guangyuan, Sichuan, China

**Keywords:** cerebral hemorrhage, antihypertensive treatment, blood pressure variability, prognosis, brain CT perfusion

## Abstract

**Background:**

Antihypertensive therapy in the acute phase of intracerebral hemorrhage (ICH) can reduce hematoma expansion. Numerous studies have demonstrated that blood pressure variability secondary to antihypertensive therapy has adverse effects on neurological outcomes, but the conclusions are diverse, and the mechanism of this occurrence is unknown. The aim of this research was to analyze the impact of blood pressure variability after antihypertensive treatment on the prognosis of patients with acute ICH, along with the possible mechanism.

**Materials and methods:**

A total of 120 patients within 20 h of onset of ICH were divided into a good prognosis group (mRS ≤ 2 points) and a poor prognosis group (mRS ≥ 3 points) according to their 90-day mRS scores. The basic patient information, NIHSS score, GCS score, mRS score at 90 days after admission, head CT examination at admission and 24 h and CTP examination at 24 h were collected from some patients. The blood pressure values of patients were collected within 24 h, and multiple blood pressure variation (BPV) parameters within 1 and 24 h were calculated.

**Results:**

(1) After excluding confounding factors such as age, whether the hematoma ruptured into the ventricle, confounding signs, amount of bleeding, edema around the hematoma, NIHSS on admission, operation or non-operation, and 24-h hematoma increment, the fourth quartile systolic blood pressure (SBP) maximum and minimum difference within 1 h [OR: 5.069, CI (1.036–24.813) *P* = 0.045] and coefficient of continuous variation (SV) within 24 h [OR: 2.912 CI (1.818–71.728) *P* = 0.009] were still independent factors affecting the 90-day mRS in ICH patients. (2) There was a negative correlation between SBP SV and CBF in terms of the difference between the contralateral side and the perihematomal region at 24 h (Rs = −0.692, *P* = 0.013).

**Conclusion:**

Blood pressure variability after antihypertensive therapy in acute ICH is one of the influencing factors for 90-day mRS in patients. A 1-h dramatic drop in SBP and 24-h SBP SV may affect the long-term prognosis of patients by reducing whole cerebral perfusion.

## Preface

Elevated blood pressure in the acute phase of cerebral hemorrhage (ICH) patients is not only the cause of bleeding but also an important clinical manifestation (Fischer et al., 2014). High blood pressure in the hyperacute phase is the main factor causing rebleeding (Murthy et al., 2016; Qureshi and Qureshi, 2018). A large number of clinical studies have confirmed that controlling the increase in blood pressure in patients with ICH in the acute phase can effectively reduce hematoma expansion and prevent the deterioration of neurological deficit symptoms and the occurrence of adverse outcomes (Yamaguchi et al., 2018; Qureshi et al., 2020). Clinical guidelines (Chinese Society of Neurology et al., 2019) domestically and overseas recommend rapid intensive antihypertensive therapy in the acute phase as level A evidence. However, antihypertensive treatment does not provide the additional benefit of preventing hematoma expansion (Qureshi et al., 2020; Moullaali et al., 2022). Several studies have shown that blood pressure variability (BPV) after antihypertensive treatment is one of the risk factors for poor prognosis of patients (Manning et al., 2014; Chung et al., 2018; Qureshi et al., 2020), but the targets and outcomes of the studies are inconsistent, and the mechanism is still unclear.

Thus, we investigated the clinical data of hospitalized patients experiencing ICH within the past 2 years to observe whether the BPV caused by acute antihypertensive treatment affects the 90 mRS of patients and to explore the possible mechanism.

## Objects and methods

### Research objects

This was a prospective study. Written approval was obtained from the Medical Ethics Committee of our hospital. In addition to the diagnostic criteria set by the 2019 Chinese Guidelines for the Diagnosis and Treatment of Intracerebral Hemorrhage as the inclusion criteria, patients also met the following conditions: age > 18 years old, onset < 24 h, SBP > 140 mmHg, neurological deficit symptoms, and imaging examination suggesting intracerebral hemorrhage. Exclusion criteria were intracerebral hemorrhage patients with onset > 24 h or admission SBP < 140 mmHg, subarachnoid hemorrhage, arteriovenous malformation, cerebrovascular amyloidosis, anticoagulant drugs, and secondary intracerebral hemorrhage due to vasculitis. Patients with severe illness and short-term death were also excluded.

### Research method

#### Clinical intervention methods

Patients with cerebral hemorrhage were given intravenous antihypertensive therapy with urapidil and maintained for 24 h after admission. The goal was to reduce the 1-h systolic blood pressure as much as possible to 120–140 mmHg or 20% lower than that at admission. Blood pressure measurements were recorded during the first 24 h and every 5 min from admission until 30 min after admission, every 10 min from 30 min to 1 h after admission, and every hour from 1 to 24 h after admission, for a total of 34 times. The blood pressure variability index (BPV) was calculated. Basic clinical data were collected, and mRS scores were followed up for 3 months. It was defined good prognosis group (mRS ≤ 2 points) and a poor prognosis group (mRS ≥ 3 points).

#### Imaging examination

A GE Light VCT 64-slice spiral CT scanner and an AW4.5 independent workstation were used as imaging equipment. Head CT at admission and 24 h after admission was completed, and simultaneous CT perfusion (CTP) examination was performed in some patients. Hematoma volume was measured using the formula of Taguda and calculated in ml. The final result was calculated as the mean of the measured values of two attending physicians. The increase in hematoma at 24 h (△ICH, ml) was calculated as the volume of hematoma at the 24-h reexamination (ml) − the volume of hematoma at admission (ml).

The selection of regions of interest for head CTP included 3–4 regions of interest (ROIs) selected 1 cm along the edge of the hematoma and the contralateral mirror region. The perfusion parameters cerebral blood flow (CBF) and cerebral blood volume (CBV) were obtained.

### Statistical analysis

Microsoft Office Professional Plus 2013 was used to access all the data of the input after completion for BPV parameters, including the average (Mean), difference (Max − Min), standard deviation (SD), coefficient of variation (CV), and continuous variation (SV).

Statistical Product and Service Solutions (SPSS) 24.0 statistical packages were used for data analysis. In univariate analysis, an independent sample *t*-test was used to compare the continuous variables with normal distribution between groups. The non-parametric Mann–Whitney *U* test was used for comparisons between groups if the distribution was not normal. Categorical variables were compared between groups using the chi-square test. Variables that were significantly different in univariate analysis were further included in multivariate binary logistic regression analysis to assess the association between the 90-day mRS score and BPV. In the comparison of continuous variables between the cerebral perfusion value and BPV, Pearson correlation analysis was used for those with a normal distribution. If the data did not conform to a normal distribution, Spearman correlation analysis was used. Statistical differences were observed at *P* < 0.05.

## Results

### Demographic and clinical characteristics

A total of 120 patients were enrolled in this study, including 71 patients in the good prognosis group and 49 patients in the poor prognosis group. The results showed (Table 1) that age, rupture into the ventricle, confounding sign, treatment method, admission NIHSS score, admission GCS score, hematoma volume, and edema range at admission, and 24-h △ICH were all different between the two groups and were related factors affecting the prognosis.

**TABLE 1:** Univariate analysis of the baseline characteristics of subjects with intracerebral hemorrhage in the good and poor prognosis groups.

	Good prognosis (*n* = 71)	Poor prognosis (*n* = 49)	*P*-value
Sex	Male 45 (63.3%)	Male 28 (57.1%)	0.491
	Female 26 (36.7%)	Female 21 (42.9%)	
Age, years	58.45 ± 12.51	63.90 ± 11.59	0.017*
Onset to treat time, hours	4.85 ± 3.956	4.27 ± 2.812	0.379
Hematoma location			0.482
Supratentorial	61 (85.9%)	45 (91.8%)	
Subtentorial	10 (14.1%)	4 (0.02%)	
Broken into ventricle			0.004*
Yes	12 (16.9%)	20 (40.8%)	
No	59 (83.1%)	29 (59.2%)	
Mixed sign			0.017*
Yes	25 (35.2%)	28 (57.1%)	
No	46 (64.8%)	21 (42.9%)	
Hypertension			0.831
No	17 (23.9%)	13 (26.5%)	
Yes, Unmedicated	19 (26.8%)	10 (20.4%)	
Yes, Intermittent medication	21 (29.6%)	14 (28.6%)	
Yes, Regular medication	14 (19.7%)	12 (24.5%)	
Diabetes			0.546
No	67 (94.4%)	44 (89.8%)	
Yes, Unmedicated	3 (4.2%)	3 (6.1%)	
Yes, Intermittent medication	0 (0%)	1 (2.0%)	
Yes, Regular medication	1 (1.4%)	1 (2.0%)	
Treatment			0.022*
Conservative treatment	69 (97.2%)	41 (83.7%)	
Evacuation of the hematoma	2 (2.8%)	8 (16.3%)	
Antihypertensive treatment			0.96
Intensive antihypertensive	33 (46.5%)	23 (46.9%)	
Non-intensive antihypertensive treatment	38 (53.5%)	26 (53.1%)	
SBP on admission, mmHg	176.2 ± 19.94	177.88 ± 21.32	0.66
DBP on admission, mmHg	105.45 ± 16.31	101.94 ± 13.70	0.22
Hematoma on admission, ml	9.5 (3.8–15.75)	25 (10.3–42.75)	0.000*
Peripheral edema on admission, ml	3.2 (1.0–7.12)	8.5 (3.53–14.77)	0.000*
NIHSS on admission	8 (4–11)	18 (13–23)	0.000*
GCS on admission	14 (13–15)	11 (8–14)	0.000*
△ICH, ml	0.97 (0.11–2.75)	2.68 (0.55–3.81)	0.047*
△PHE, ml	0.77 (0.0–3.36)	1.45 (0.0–8.99)	0.160

Mean ± SD; Median (IQR), Median (interquartile spacing); PHE, Peripheral edema; △ICH, Hematoma enlargement at 24 h; △PHE, the enlargement of peripheral edema at 24 h. *The difference was statistically significant at *P* < 0.05.

### Effects of blood pressure variation at different periods after antihypertensive treatment on the 90-day neurological outcome in patients with acute intracerebral hemorrhage

The effect of BPV at different time periods (within 1 and 24 h) on clinical outcomes was evaluated. The results showed (Table 2) that the SBP max-min, SD and CV of the two time periods and 24-h SBP SV were different between the good prognosis group and the poor prognosis group (*P* < 0.05). The 1-h SBP SV (*P* < 0.1) was included in the multivariate analysis.

**TABLE 2:** The effects of BPV on the prognosis of ICH patients.

	Good prognosis (*n* = 71)	Poor prognosis (*n* = 49)	*P*-value
1 h SBP			
Mean	160.06 ± 13.90	160.98 ± 47.34	0.723
Max-Min	36.25 ± 16.27	43.31 ± 21.88	0.023*
SD	12.02 ± 5.98	14.72 ± 7.39	0.029*
CV	0.75 ± 0.037	0.09 ± 0.046	0.034*
SV	28.56 (16.44–55.33)	41.27 (21.03–90.72)	0.051
24 h SBP			
Mean	147.23 ± 11.72	149.00 ± 9.88	0.388
Max-Min	62.99 ± 17.85	70.73 ± 21.66	0.034*
SD	15.78 ± 4.75	18.16 ± 5.65	0.014*
CV	0.11 ± 0.034	0.12 ± 0.039	0.031*
SV	67.89 (49.11–96.34)	89.61 (64.38–134.46)	0.009*

$\bar{\text{x}}$ ± s, Mean ± SD; Median (IQR), Median (interquartile spacing). *The difference was statistically significant at *P* < 0.05.

There was no significant difference in diastolic blood pressure variation parameters between the two groups (*P* > 0.05, data not listed).

### The interquartile blood pressure variation parameters on the adverse prognosis of intracerebral hemorrhage patients

**FIGURE 1:**
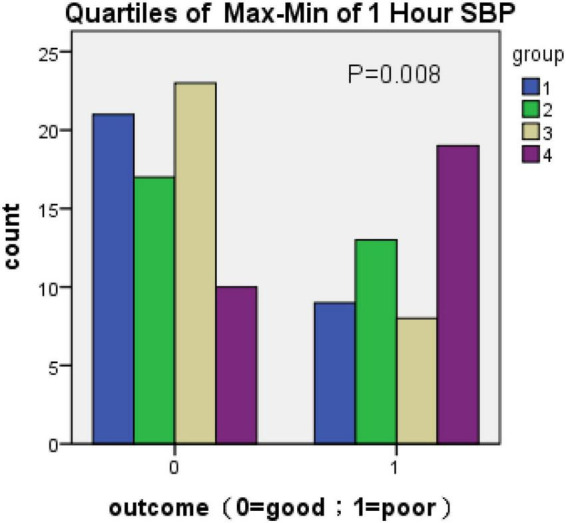
Quartiles of Max-Min of 1-h SBP (outcome: 0 = good; 1 = poor).

**FIGURE 2:**
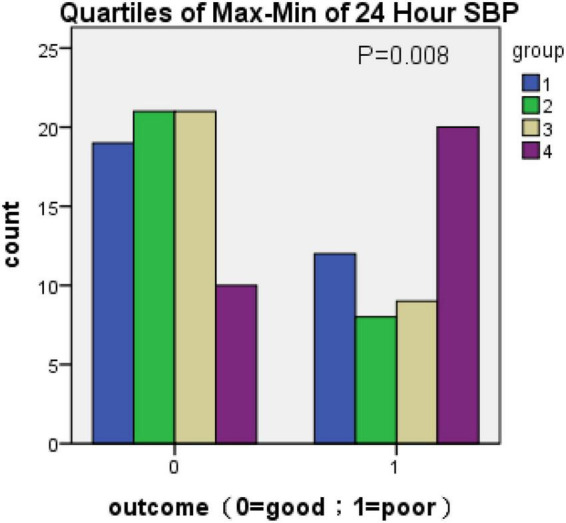
Quartiles of Max-Min of 24-h SBP (outcome: 0 = good; 1 = poor).

**FIGURE 3:**
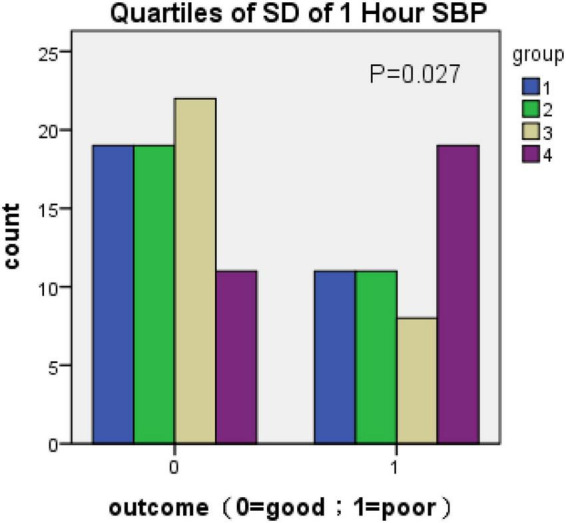
Quartiles of SD of 1-h SBP (outcome: 0 = good; 1 = poor).

**FIGURE 4:**
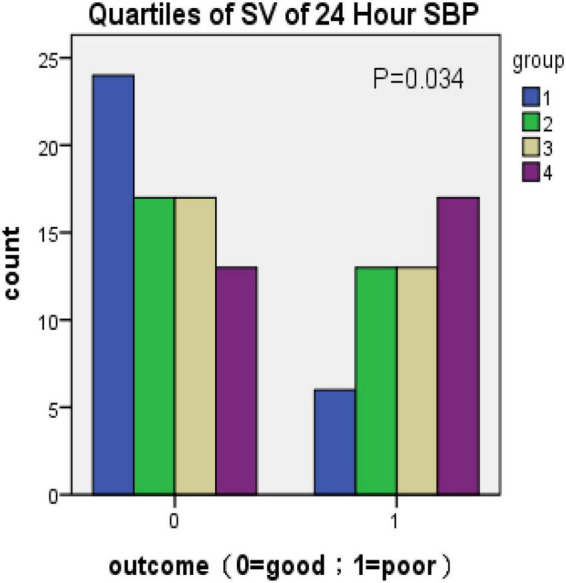
Quartiles of SV of 24-h SBP (outcome: 0 = good; 1 = poor).

Because there was no statistical difference in the above parameters obtained according to Table 2 in the multivariate analysis, the parameters of BPV with statistically significant differences were divided into four groups (q1–q4) according to the quartile as the boundary value. The R × C chi-square test was used to compare the incidence of poor prognosis in each group. The correlation between BPV and poor prognosis was analyzed by quartile group comparison. The results showed that 1 h SBP Max-Min (χ2 = 11.746, *P* = 0.008) 24 h SBP Max-Min (χ2 = 11.908, *P* = 0.008), 1 h SBP SD (χ2 = 9.21, *P* = 0.027), 24 h SBP SV (χ2 = 8.658, *P* = 0.034), suggesting that the above variables were correlated with prognosis (Figures 1–4 below for details).

### Multivariate analysis of prognostic value of parameters in patients with intracerebral hemorrhage

Multivariate binary logistic regression analysis was used to evaluate the effect of BPV parameters in different time periods and different intervals on the poor prognosis of neurological function in patients with acute intracerebral hemorrhage. The results showed that the 1-h Max-Min and 24-h SBP SV q4 regions were significantly associated with 90-day neurological outcomes (Table 3).

**TABLE 3:** Multivariate logistic regression analysis of the prediction of different interval BPV parameters for poor prognosis.

	1-h SBP (MAX-MIN)		1-h SBP SD		24-h SBP (MAX-MIN)		24-h SBP SV	
				
	OR (CI)	*P*	OR (CI)	*P*	OR (CI)	*P*	OR (CI)	*P*
q1	–	0.232	–	0.301	–	0.157	–	0.08*
q2	2.41 (0.557–10.429)	0.239	0.92 (0.213–3.97)	0.911	0.392 (0.075–2.041)	0.266	4.674 (0.797–27.424)	0.088
q3	1.77 (0.333–9.417)	0.503	0.8 (0.167–3.85)	0.784	1.07 (0.262–4.368)	0.925	3.457 (0.768–29.189)	0.094
q4	5.069 (1.036–24.813)	0.045*	3.087 (0.684–13.93)	0.143	3.008 (0.67–13.498)	0.151	2.912 (1.818–71.728)	0.009*

Multiple independent variables including age, rupture into ventricle, confounding sign, first blood loss, NIHSS score, GCS score, and △ICH were adjusted. q1–q4 interquartile range. *The difference was statistically significant at *P* < 0.05.

### Effect of blood pressure variability on cerebral perfusion after antihypertensive treatment

**FIGURE 5:**
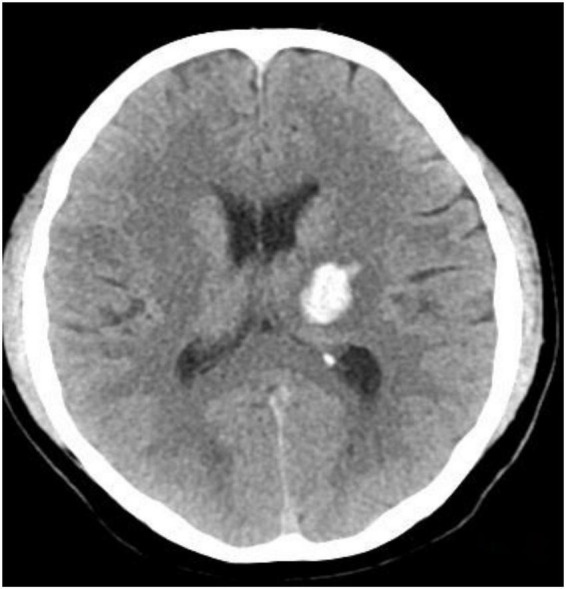
The hematoma area.

**FIGURE 6:**
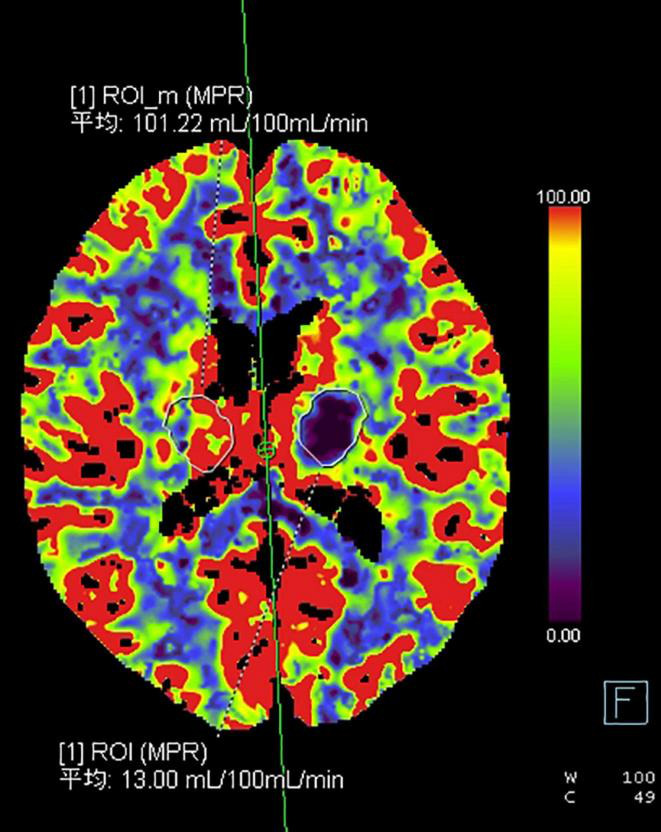
The hematoma marginal area on the CBF map.

**FIGURE 7:**
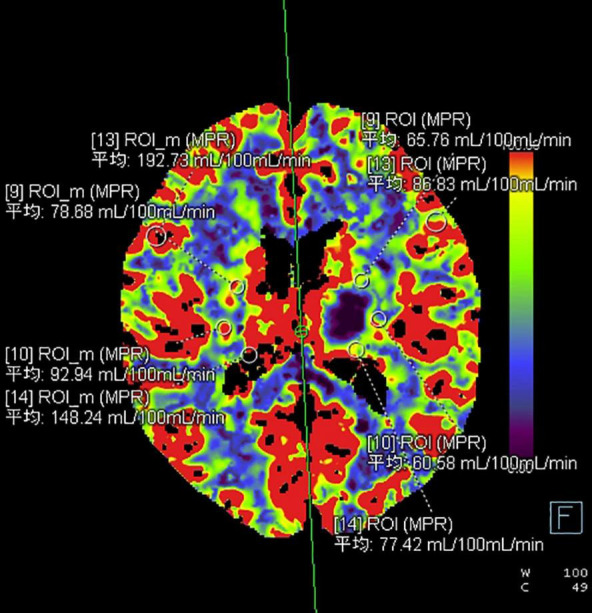
The ROI and CBF values of the hematoma marginal area, cortical area, and corresponding mirror area.

**FIGURE 8:**
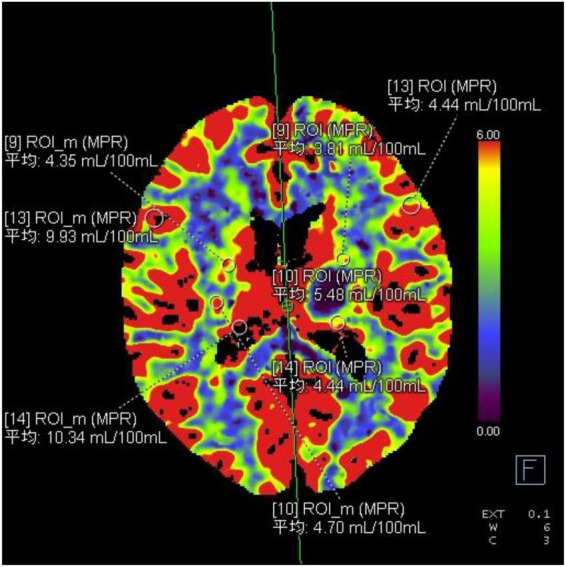
The CBV values in each ROI area.

Twenty-two patients were randomly selected to undergo a 24-h CT (Figure 5) scan and simultaneous perfusion (CTP) examination. CTP (Figures 6–8) showed a significant reduction in perfusion in the core area and periphery of the hematoma, as well as a significant reduction in CBF and CBV.

The Spearman correlation analysis of blood pressure variation parameters and cerebral perfusion parameters at each time period showed that the difference in CBF between the perihematoma and contralateral mirror area (contralateral to affected-side blood flow) was negatively correlated with 24 h SBP SV, while there was no statistically significant difference in the correlation analysis of CBF and CBV around the hematoma or CBF and CBV in the mirror area (Table 4).

**TABLE 4:** Spearman correlation analysis of the relationship between the 24-h SBP SV and the CBF difference and CBV difference.

Variables	Coefficient r	*P*-value
Healthy side CBF	–0.19	0.713
Healthy side CBV	–0.063	0.846
Affected side CBF	–0.049	0.880
Affected side CBV	0.021	0.948
CBF difference	–0.692	00.013*
CBV difference	–0.035	0.914

Cerebral blood flow (CBF) difference = healthy side CBF − affected side CBF; cerebral blood volume (CBV) difference = healthy side CBV − affected side CBV.

A 43-year-old male presented with 3 h of numbness and immobility in the right limb. Head CT (Figure 5) showed cerebral hemorrhage in the left basal ganglia area. CTP (Figure 6) showed the hematoma area; CTP (Figure 7) showed the ROI and CBF values of the hematoma marginal area, cortical area, and corresponding mirror area; CTP (Figure 8) showed the CBV values in each ROI area.

## Discussion

In the past 10 years, a number of large-scale international clinical studies have confirmed that early intensive antihypertensive therapy can effectively control hematoma expansion and bring more obvious blood pressure fluctuations (blood pressure variability, BPV), which may have adverse effects on patients with ICH.

Chung et al. (2018) analyzed the Fast-Mag Trial (Field Administration of Stroke Therapy-Magnesium), a study of prehospital care. The patients with obvious blood pressure variability in the hyperacute phase (0–6 h) and acute phase (0–24 h) had a poor prognosis rate (mRS 3–6 scores) of 69.9%. Among them, SD, CV, and SV were all related to poor prognosis in the acute phase and hyperacute phase. The OR of the highest quintile was 3.73, the OR of the coefficient of variation was 4.78, and the OR of the SV was 3.39. Conclusion: blood pressure variability in the acute phase of ICH in the prehospital emergency room is an independent risk factor for poor prognosis, and it is also one of the targets of clinical treatment. Qureshi et al. (2020) retrospectively analyzed a clinical multicenter blood pressure control study from 2011 to 2015. The results showed that 228 patients with ICH whose initial blood pressure was equal to or greater than 220 mmHg were treated with intensive blood pressure control or standard blood pressure control. The proportion of neurological deterioration within 24 h in the intensive treatment group was higher than that in the non-intensive treatment group (15.5 vs. 6.8%; RR 2.28 [95% CI, 1.03–5.07]; *P* = 0.04). There was no significant difference in mortality or severe disability rate between the two groups (39.0 vs. 38.4%; RR 1.02 [95% CI, 0.73–1.78]; *P* = 0.92). This suggests that early and rapid lowering of blood pressure may be harmful. Manning et al. (2014) conducted a retrospective analysis of the INTERACT2 study, which showed that the maximum systolic blood pressure was in the hyperacute phase (24 h), and SD was a marker of death in the acute phase (2–7 days) and poor prognosis at 90 days (OR = 1.41, *P* = 0.016; OR = 1.57, *P* = 0.012). It is suggested that smooth and sustained control of blood pressure is needed in the ultra early stage. Liu W. et al. (2021) included 7 randomized controlled prospective studies with 5,201 participants in their meta-analysis. Increased SBP variability was associated with an increased risk of poor functional outcomes: The results are SD (OR: 1.38, *P* = 0.001), CV (OR: 1.98, *P* = 0.017), SV (OR: 1.30, *P* = 0.006), and the effect of SBP variability on the risk of poor functional outcomes in ICH patients was affected by country, study design, mean age, stroke type, outcome definition, and study quality.

The aim of our study was to observe the 90-day prognosis of patients with ICH after antihypertensive treatment. The results showed that after excluding other factors that clearly affect the prognosis of ICH, the top quartile of SBP 1-h max-min and 24-h SBP SV was still an independent risk factor for ICH patients with a poor prognosis at 90 days. We hypothesized that BPV affects clinical outcomes through the following mechanisms: 1. Destroy perihematomal perfusion. 2. Change the microcirculation of the whole brain.

Whether early lowering of blood pressure results in lower early cerebral perfusion remains controversial. On the basis of INTERACT1 and ATACH experiments, studies such as ICH ADAPT suggest that there is no significant correlation between the magnitude of blood pressure reduction and the relative cerebral blood flow around the hematoma (Powers et al., 2001). Moreover, the SBP in a small number of ICH patients is controlled at the target value within 24 h. The blood flow of the brain tissue around the hematoma can be maintained by autoregulation (Gould et al., 2014; Tanaka et al., 2014; Tamm et al., 2016). With the progress of INTERACT2 and ATACH2 experiments, Gould et al. (2014) demonstrated that the blood perfusion around the hematoma, perihematoma tissue area and watershed tissue area of early ICH (<2 h) was below the ischemic threshold by CTP, but there was no significant correlation between intensive hypotension and hypoperfusion area. In Our study, CTP of some patients was performed at the same time as reexamination of head CT 24 h after admission, and decreased CBF and CBV around the hematoma was found. Table 3 showed that only the 1-h SBP Max-Min in the fourth quartile was associated with poor outcome, suggesting that the amplitude of blood pressure reduction is a factor of functional recovery. Kuang et al. (2015) showed the perihematomal ischemic injury in acute supratentorial large hematoma. Also found a synchronous decreased CBF and CBV around hematoma suggesting microcirculation compression. As a result, the autoregulation ability of cerebral perfusion in this region was weakened or disappeared. The sharp short-term drop in blood pressure again causes perihematoma hypoperfusion to aggravate neurological injury.

In addition, the above studies were limited to the hyperacute phase. Regarding sustained blood pressure reduction, a recent study by Morotti et al. (2022) showed that cerebral perfusion around the hematoma gradually decreased with the development of the disease, and 7-day CBF < 20 ml/100 g/min was shown to be an independent predictor of poor functional outcome (OR: 2.45, 95% CI 1.08-5-54, *p* = 0.032).

To further explore the relationship between BPV and cerebral perfusion, we performed correlation analysis on the related variation parameters affecting the poor prognosis of patients and ultimately showed that there was a negative correlation between 24-h SBP SV and the CBF difference between the healthy side and the affected side in patients with ICH; that is, the larger the 24-h SBP SV was, the smaller the CBF difference value. It is speculated that the reason for this phenomenon is that the cerebral perfusion around the hematoma loses its autoregulation ability early due to compression by the hematoma and edema and is less affected by blood pressure fluctuations.

Previous studies (Rickards and Tzeng, 2014; Kario, 2016) have demonstrated that cerebral hemodynamic variables (blood pressure and cerebral blood flow) have physiologically protective characteristics and can cause extensive damage to organ function. These apparently different outcomes may lie in the time scale of hemodynamic variability; short time-scale variability appears to be brain protective, whereas medium- and long-term fluctuations are associated with primary and secondary end-organ dysfunction. Blood pressure fluctuations can damage vascular endothelium and blood-brain barrier, increase small vessel resistance, and reduce brain cell metabolism (Liu N. et al., 2021) resulting in decreased CBF. The change is systemic and as to healthy tissue is particularly significant compared to impaired blood circulation around the hematoma.

There was no significant difference in cerebral perfusion parameters between healthy brain areas and surrounding hematoma, which may be related to the small sample size or the fact that the difference did not reach statistical significance. Unfortunately, although 1-h SBP max-min is a poor prognostic factor in ICH patients, its role in cerebral perfusion is not clear due to the short time from admission to hospitalization.

## Conclusion

The 1-h quartile difference in SBP and 24-h SBP SV are independent risk factors for poor prognosis in patients with intracerebral hemorrhage. Excessive hypotension and secondary continuous blood pressure fluctuations cause secondary brain injury through whole-brain perfusion and then aggravate the neurological impairment of patients with cerebral hemorrhage.

## Data availability statement

The original contributions presented in this study are included in the article/supplementary material, further inquiries can be directed to the corresponding author.

## Ethics statement

The studies involving human participants were reviewed and approved by the Ethics Committee of Affiliated Hospital of North Sichuan Medical College 2020ER194-1. The patients/participants provided their written informed consent to participate in this study. Written informed consent was obtained from the individual(s) for the publication of any potentially identifiable images or data included in this article.

## Author contributions

XS: project design, declaration, experiment coordinating and organization, translation, and revision. XJ: data collection, analysis, and draft. QM, QY, and TC: clinical cases. GJ: project administration. All authors contributed to the article and approved the submitted version.
